# Multimodal Object Classification Models Inspired by Multisensory Integration in the Brain

**DOI:** 10.3390/brainsci9010003

**Published:** 2019-01-02

**Authors:** Rajesh Amerineni, Resh S. Gupta, Lalit Gupta

**Affiliations:** 1 Department of Electrical and Computer Engineering, Southern Illinois University, Carbondale, IL 62901, USA; rajeshamerineni@siu.edu; 2 Vanderbilt Brain Institute, Vanderbilt University, Nashville, TN 37232, USA; resh.s.gupta@vanderbilt.edu

**Keywords:** multisensory integration, multimodal object classification, feature and decision integration

## Abstract

Two multimodal classification models aimed at enhancing object classification through the integration of semantically congruent unimodal stimuli are introduced. The feature-integrating model, inspired by multisensory integration in the subcortical superior colliculus, combines unimodal features which are subsequently classified by a multimodal classifier. The decision-integrating model, inspired by integration in primary cortical areas, classifies unimodal stimuli independently using unimodal classifiers and classifies the combined decisions using a multimodal classifier. The multimodal classifier models are implemented using multilayer perceptrons and multivariate statistical classifiers. Experiments involving the classification of noisy and attenuated auditory and visual representations of ten digits are designed to demonstrate the properties of the multimodal classifiers and to compare the performances of multimodal and unimodal classifiers. The experimental results show that the multimodal classification systems exhibit an important aspect of the “inverse effectiveness principle” by yielding significantly higher classification accuracies when compared with those of the unimodal classifiers. Furthermore, the flexibility offered by the generalized models enables the simulations and evaluations of various combinations of multimodal stimuli and classifiers under varying uncertainty conditions.

## 1. Introduction

The main goals of this multidisciplinary machine learning and neuroscience collaboration are (a) to formulate multimodal classifier models inspired by multisensory integration in the brain, and (b) to demonstrate that the resulting classifiers improve object recognition through the integration of semantically congruent unimodal stimuli. A generalized unimodal classification model is introduced, and two purely feed-forward multisensory multimodal models, namely, the feature-integrating (FI) and the decision-integrating (DI) models, are derived from this unimodal model. The two models differ in the type of information that is integrated. The FI model, inspired by multisensory integration in the subcortical superior colliculus (SC), combines unimodal features which are subsequently classified by a multimodal classifier. In the DI model, inspired by integration in primary cortical areas, the unimodal stimuli are classified independently by unimodal classifiers and the unimodal decisions are combined and classified by a multimodal classifier.

The key contribution of this study is the systematic development of the two multimodal classification models with parameters that can be manipulated to simulate (a) the weighted influence of each stimulus in the integration process, (b) different representations of the stimuli in the modalities, (c) mechanisms for combining different forms of unimodal information, and (d) varying degrees of uncertainty in the environment during stimuli integration. Supporting contributions include the design of experiments to demonstrate the versatility of the models and the improvement in performance by combining unimodal stimuli with various attenuation levels in varying degrees of noise. Specifically, each model is developed using artificial neural network and statistical classifiers to show that the classification models are not restricted to any particular type of classifier. The experiments involve the classification of auditory and visual representations of ten digits; however, the models can combine stimuli from other modalities and the number of modalities can be greater than two. 

### 1.1. Multisensory Integration

**Figure 1:**
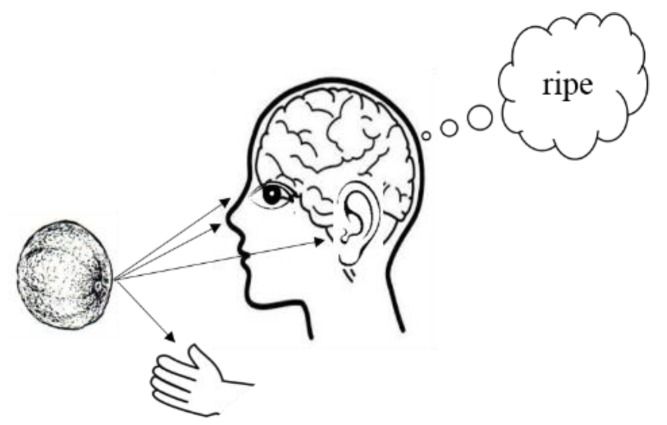
The senses involved in deciding whether a cantaloupe is ripe.

Humans have multiple senses, including the primary senses of sight (vision), hearing (audition), taste (gustation), smell (olfaction), and touch (somatosensation). The secondary senses include perception of temperature (thermoception), kinesthesia (proprioception), pain (nociception), balance (equilibrioception), and vibration (mechanoreception) [1]. Among the numerous fascinating and complex operations performed by the brain, one of the most important is “multisensory integration,” which is the brain’s ability to combine information from different sensory modalities to robustly and coherently perceive the external environment [2,3,4,5]. The three rules that govern multisensory integration are the spatial, temporal, and inverse effectiveness rules, which state that multisensory integration is enhanced (more likely or stronger) when stimuli occur at approximately the same location, at approximately the same time, and when the unimodal stimuli in the set evoke relatively weak responses, respectively [5]. Multisensory integration enhances the detection of external stimuli, facilitates object recognition, resolves ambiguities and conflicts, and decreases reaction times [5,6]. As illustrated in the simplified diagram in Figure 1, the simple act of deciding whether a cantaloupe is ripe or unripe can involve examining the color (vision), tapping to detect the sound for hollowness (audition), feeling the skin netting (somatosensation), and smelling to detect the scent (olfaction).

Many studies have focused on the integration of auditory and visual stimuli. Originally, the established view posited that vision dominates multisensory perception of the world, however, it is now understood that visual perception can actually be manipulated by other sensory modalities [7]. These effects are well-demonstrated in conflicts arising from audio-visual integration [8]. As an example, visual processing dominates auditory processing during spatial processing (e.g., spatial localization of audio-visual stimuli); therefore, when there is conflict between the visual and auditory signal, the response will be influenced mostly by the visual signal. This phenomenon is clearly observed in the Ventriloquist effect [9,10], which refers to the ancient art of making one’s voice appear to come from elsewhere. When spatially localizing audio-visual stimuli, vision dominates audition when visual localization is good. However, under situations where visual stimuli are severely blurred (poorly localized), audition dominates vision [10]. In contrast, during temporal processing, audition dominates vision; this can be observed in the sound-induced illusory flash effect [7]. In this effect, when a single visual flash is accompanied by multiple auditory beeps, the single flash is incorrectly perceived as multiple flashes [7]. Dominance in audio-visual speech perception is also famously observed in the McGurk effect [11], which demonstrates the effect of vision on speech perception. In McGurk and MacDonald’s original study, a spoken /ba/ syllable dubbed onto a visual presentation of /ga/ was reported as /da/ on a majority of trials [12].

Several models of multisensory integration at different cortical levels have been reported, and most are based on integration in the subcortical SC and integration in primary cortical areas. In the SC integration model, SC neurons are multisensory and have a receptive field (RF) for each sensory modality [13,14,15]. The RFs partially overlap in sensory space, receive converging unisensory information, and combine this information in an appropriate way. The earlier models of integration in primary cortical areas assume that unimodal stimuli are processed separately in the primary cortices and are then combined in higher-order association areas in the brain [16,17].

Over the past decades, various approaches have been used to explain and demonstrate, at the neural level, many features of multisensory integration [5,6,18,19,20]. Notable neural network-based contributions, among many others, include the detailed mathematical description of a three-neural network model to mimic various responses to multisensory stimuli [18]. A Bayesian framework, which provides a general theory for multisensory integration, is another effective approach that has been developed, and numerous studies have been conducted to demonstrate the validity of this approach with respect to multimodal enhancement [4,19,20].

### 1.2. Scope of Research

We emphasize that our goal is not aimed at contributing to the wealth of research related to multisensory integration in the brain, but to demonstrate that the two proposed multimodal classification systems are able to emulate, at the systems (input-output) level, some properties related to multisensory object recognition. Specifically, we focus on the following interpretation of the principle of inverse effectiveness: combinations of weakly effective unimodal stimuli produce greater “responses” when compared with the response of the most effective stimulus in the set [5,21]. In the context of object classification, “responses” can be replaced by “classification accuracies.” Consequently, our goal is to show that object classification can be enhanced by combining unimodal stimuli. For demonstration purposes, we focus on the improvement in object classification through the integration of bimodal information extracted from the auditory and visual modalities. However, the generalized formulations of the multimodal classification models enable the integration of information from other modalities. Furthermore, the number of modalities can exceed two.

As noted earlier, in order to demonstrate the invariance of each model to any particular type of classifier, the models are developed using artificial neural network and statistical classifiers. Here too, we emphasize that our goal is not aimed at developing new and improved machine learning classification algorithms, nor is it aimed at determining the best set of features for classifying multimodal stimuli. Instead, our clear goal is to select a classifier and a feature set for each sensory modality, combine these unimodal classifiers into multimodal classification systems, and compare the performance of the resulting multimodal classifiers against the performance of the unimodal classifiers. We select two well-known classifiers: the multilayer perceptron (MLP) neural network classifier and the maximum a posteriori (MAP) classifier; however, it should be clear that other classifiers such as deep-learning neural networks, support vector machines, logistic regression, and decision-tree classifiers can be used in the models.

A preliminary 4-page version of this study describing the MLP implementations of the models and performance in noise was presented at BHI2018 [22]. This 14-page expanded version is a more complete study aimed at demonstrating the versatility of the unimodal and multimodal models by including (a) MAP implementations, (b) additional experiments and analyses in the presence of stimulus noise and amplitude attenuations, (c) a detailed discussion of the results, and (d) suggestions on how the models can be used to demonstrate the spatial and temporal rules. Also included is a more detailed introduction with additional references.

The proposed FI and DI multimodal classification systems are related to ensemble and data fusion classifiers. Typically, ensemble classifiers combine classifiers whereas data fusion classifiers combine data from multiple sensors [23,24,25,26]. These classifiers do not attempt to emulate multisensory integration in the brain, but are aimed at improving the overall classification accuracy by combining diverse classifiers and/or by exploiting complementary information from different sensors. However, the FI and DI models formulated in this paper have several notable features pertaining to multisensory integration, including the ability to tailor the classification systems for each modality and across modalities. For example, (a) the unimodal classifiers can differ across modalities to account for differing classification mechanisms in the modalities, (b) the feature sets can differ across modalities to account for differing “internal mental representations” extracted in the modalities, and (c) the type of noise and attenuation levels can differ across modalities to account for different types of variability in the multimodal stimuli. 

The FI and DI multimodal models differ quite significantly in their structures, how information is defined, and the manner in which multimodal information is integrated. The main difference between the structures is that the FI model has a single classifier, and the DI model has multiple classifiers: one for each stimulus modality. The information integrated in the FI model is the set of features extracted from the unimodal stimuli, whereas the information integrated in the DI model is the set of decisions of the unimodal classifiers. The following sections describe the formulations of the unimodal and the two multimodal models for the multiclass classification of unimodal and multimodal stimuli. For each model, the generalized formulation is described first and the MLP and MAP formulations follow. The stimuli classes are represented by $\omega_{k},k=1,2,\ldots ,K$ and the sensory stimuli are represented by $S_{j},j=1,2,\ldots ,J,$ where K and *J* are the number of stimulus classes and the number of sensory modalities, respectively.

## 2. Unimodal Classification Model

**Figure 2:**
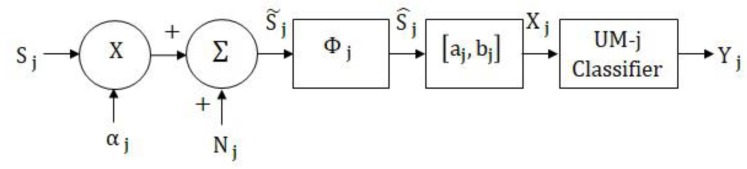
Block diagram of the unimodal classification system for modality *j*.

The generalized classification model for a single sensory modality is illustrated in Figure 2, in which the unimodal classifier for modality j is represented by UM-j. This systems-level model assumes that unimodal stimuli are processed separately in their respective primary channels (primary cortical areas), and there is no cross-modal interaction. Although unrealistic, this model is described in detail and implemented for the purpose of comparing the performances of the multimodal classifiers against the unimodal classifiers. Furthermore, this model is an integral part of the proposed multimodal classification systems which contain the same uncertainty parameters (noise and attenuations).

In this model for modality $j,\;S_{j}$ is the $d_{j}$-dimensional input sensory signal, $\alpha_{j}$ is the signal attenuation (weighting) factor, $N_{j}$ is the random noise added to the signal, $\Phi_{j}$ is the feature generating matrix, $\left[a_{j},b_{j}\right]\;$ is the normalizing interval, $X_{j}$ is the classifier input, and $Y_{j}$ is the system output. The dominance (amplitude or strength) of the input sensory signal can be adjusted by $\alpha_{j}$, which takes values in the interval [0,1], where, zero corresponds to infinite attenuation (zero amplitude or signal absent) and one corresponds to zero attenuation (full amplitude or strength). During the training phase (parameter estimation), $\alpha_{j}$ is set to 1 but can be varied during testing to evaluate the performance as a function of stimulus attenuations.

The external noise signal $N_{j}$ is assumed to be uncorrelated with the unimodal signal and is also assumed to be multivariate Gaussian with zero mean and covariance $\Psi_{j}$, that is, $N_{j}\sim G\left(0,\Psi_{j}\right).$ Features are generated by multiplying ${\tilde{S}}_{j}$ with the $D_{j}\times d_{j}$ transformation matrix $\Phi_{j}$ such that the *D_j_* features are linear combinations of the elements of vector ${\tilde{S}}_{j}$. Because of its simplicity, data independence (fixed basis vectors), and information packing capabilities, the discrete cosine transform (DCT) is selected for $\Phi_{j}\;j=1,2,\ldots ,J,$ [27]. The feature vector may be regarded as an “internal or mental representation” of the stimulus. The features are normalized to take values in the interval $\left[a_{j},b_{j}\right]$ using a linear transformation of the form$\;m_{j}{\hat{S}}_{j}+c_{j}$, where, $m_{j}$ and $c_{j}$ are the slope and intercept of the line connecting $\left({\hat{S}}_{j,min},a_{j}\right)$ and $\left({\hat{S}}_{j,max},b_{j}\right)$, and where ${\hat{S}}_{j,min}$ and ${\hat{S}}_{j,max}$ are the minimum and maximum values of ${\hat{S}}_{j}$, respectively. In the final step, the unimodal classifier determines the K-dimensional vector $Y_{j}$, which has the information needed, such as posterior probabilities or discriminants, to assign the sensory signal to one of the K stimulus classes. 

### 2.1. Unimodal MLP Network Classifier

As noted in the introduction, the neural network selected for the generalized multiclass classification problem is the MLP. The network is fully interconnected between layers. The network determines a mapping $f:X_{j}{}^{D_{j}}\to Y^{K}$, using the backpropagation training algorithm, between input $X_{j}$ of dimension $D_{j}$ and output *Y* of dimension K. In general, the output of a fully interconnected MLP with M layers (layer 1 is the input), expressed as a composite of functions, is given by

$$Y=f\left(X_{j}\right)=f_{M}\left(W_{M-1}^{T}\ldots f_{3}\left(W_{2}^{T}f_{2}\left(W_{1}^{T}X_{j}+\beta_{2}\right)+\beta_{3}\right)\ldots +\beta_{M}\right),$$

where, $f_{m}$ is the activation function in layer m, m≠1, $\beta_{m}$ is the bias into layer m, m≠1, and $W_{m},\;m\neq M,$ is the $N_{m}\times N_{m+1}$ interconnection weight matrix between layers m and (m+1) which have dimensions $N_{m}$ and $N_{m+1}$, respectively.

For the multiclass classification problems considered in this study, we select the sigmoidal activation function for the intermediate hidden layers, the softmax activation function for the output layer, and the cross-entropy for the loss-function. Therefore, the hidden layer outputs are given by $f_{m}\left(q_{i}\right)=\frac{1}{1+e^{-q_{i}}},\;m=2,\ldots ,\left(M-1\right)$, and the network outputs are given by

$$f_{M}\left(q_{k}\right)=y\left(k\right)=\frac{e^{q_{k}}}{\sum_{k=1}^{K}e^{q_{k}}},\;k=1,2,\ldots ,K,$$

where, $q_{n}$ is the weighted sum of the inputs into a neuron n in the respective layer and y(k) is the $k^{th}$ output of the network. If *t_k_* is the target of y(k), k=1,2,…,K, the cross-entropy cost function is given by

$$E=-\overset{K}{\underset{K=1}{\sum }}t_{k}\log(y\left(k\right)),\;\mathrm{where},$$

$$t_{k}=\{\begin{array}{c} 1\;if\;X_{j}\;\epsilon \omega_{k}\\ 0\;otherwise \end{array}.$$

Using the maximum response rule, the neural network assigns the input stimulus to the class associated with the output that yields the largest value. Because the softmax activation function is selected, the outputs y(k),k=1,2,…,K, can be regarded as estimates of the posterior probabilities. Consequently, a test feature X can be assigned to class $\omega_{k}$ if

$$y\left(k\right)=P(\omega_{k}/X_{j})>y\left(i\right)=P(\omega_{i}/X_{j}),\;\mathrm{for}\;\mathrm{all}\;i\neq k.$$

### 2.2. Unimodal MAP Classifier

The statistical classifier selected is the MAP classifier, which is the Bayes classifier for a 0–1 loss function. For a given test feature vector $X_{j}$, the MAP classifier determines all K posterior probabilities and decides in favor of the class which yields the highest posterior probability. If the class-conditional probability density of $X_{j}$ under $\omega_{i}$ is $P\left(X_{j}/\omega_{i}\right)$ and the prior probability of class $\omega_{i}$ is $P\left(\omega_{i}\right)$, the posterior probability of class $\omega_{i}$ given *X_j_* is determined from

$$P(\omega_{i}/X_{j})=\frac{P\left(X_{j}/\omega_{i}\right)P\left(\omega_{i}\right)}{\sum_{i=1}^{K}P\left(X_{j}/\omega_{i}\right)P\left(\omega_{i}\right)},\;i=1,2,\ldots ,K.$$

The MAP classifier assigns $X_{j}$ to the class with the largest posterior probability. That is, $X_{j}$ is assigned to class $\omega_{k}$ if

(1) $$P(\omega_{k}/X_{j})>P(\omega_{i}/X_{j}),\;\mathrm{for}\;\mathrm{all}\;i\neq k$$

Equivalently, the classifier can be expressed in terms of discriminant functions, and $X_{j}$ is assigned to class $\omega_{k}$ if

$$g_{k}\left(X_{j}\right)>g_{i}\left(X_{j}\right),\;\mathrm{for}\;\mathrm{all}\;i\neq k,$$

where,$\;g_{i}\left(X_{j}\right)=lnP(X_{j}/\omega_{i})+lnP\left(\omega_{i}\right)$ is the discriminant function of $X_{j}$ under class $\omega_{i}$.

In order to develop the MAP classifier in the unimodal classification system shown in Figure 2, the conditional density function $P\left(X_{j}/\omega_{k}\right),\;k=\;1,2,\ldots ,K,$ has to be derived systematically. As noted earlier, the noise vector $N_{j}$ is $G\left(0,\Psi_{j}\right)$ and *α_j_* is set to 1 during the parameter estimation phase. Given that all processing steps are linear, it can be shown that signals at various stages and their corresponding probability density functions $P\left(•/\omega_{k}\right),\;k=\;1,2,\ldots ,K$, are given by

$${\tilde{S}}_{j/k}=(S_{j/k}+N_{j})\sim G\left(S_{j/k},\Psi_{j}\right),$$

$${\hat{S}}_{j/k}=\Phi_{j}\left({\tilde{S}}_{\frac{j}{k}}\right)\sim G\left(\Phi_{j}S_{\frac{j}{k}},\Phi_{j}\Psi_{j}\Phi_{j}{}^{T}\right),$$

$$X_{j/k}=\left(m_{j/k}{\hat{S}}_{j/k}+c_{j/k}\right)\sim G\left(m_{j/k}\Phi_{j}S_{j/k}+c_{j/k},m_{j/k}{}^{2}\Phi_{j}\Psi_{j}\Phi_{j}{}^{T}\right).$$

Because the classifier outputs are the posterior probabilities $P(\omega_{i}/X_{j}),\;i=1,2,\ldots ,K,$ the rule in Equation (1) can be used to assign a test vector $X_{j}$ to the class yielding the highest posterior probability.

The next two sections describe the FI and DI multimodal classification models which are derived from the unimodal model. The initial processing steps for each modality in both models are identical to the processing steps of the unimodal model. For consistency, and to facilitate the descriptions, the implementations that follow assume that each model contains the same uncertainty parameters and feature generating matrices. Furthermore, it is assumed that the unimodal classifiers in the DI model belong to the same family of classifiers (neural network or MAP). However, it is important to note that uncertainty parameters and feature matrices can differ across the modalities and the DI classification system can contain a mixture of neural network, statistical, or other forms of unimodal classifiers.

During the training phase, it is assumed that the multimodal stimuli presented to the system are semantically congruent, have no attenuations, and are temporally aligned. The attenuation factor $\alpha_{j}$ can be varied during testing to evaluate the performance as a function of varying attenuation levels across modalities. For example, in cases where visual processing dominates auditory processing, the attenuation factor for the visual sensory signal can be set to 1 while the factor for the auditory sensory signal is set to a fraction that is proportional to the relative lack of effectiveness of the auditory signal. The attenuation levels can also be adjusted to simulate the effects of varying conditions, such as visual dominance during daylight and auditory dominance at night. The random noise can be statistically different across modalities, and the level of noise that is added to each stimulus modality can be adjusted to determine the effects of increasing noise in one or more modalities.

## 3. Feature-Integrating Multimodal Classification Model

**Figure 3:**
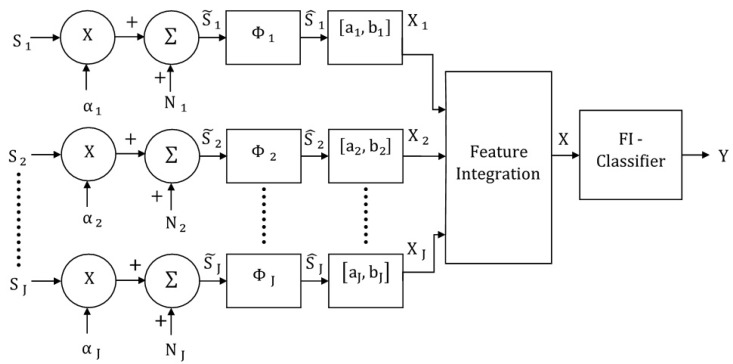
Block diagram of the feature-integrating multimodal classification system.

The generalized feature-integrating model for the multimodal classification system is shown in Figure 3, in which the feature-integrating classifier is represented by FI. The system combines the normalized unimodal feature vectors through concatenation, and the resulting vector is the input to a multimodal classifier which determines the class of the multimodal stimulus presented to the system. This model can be regarded as a systems-level representation of a pure feedforward system, which is often used to simulate activity of multisensory neurons in the superior colliculus [14,15]. For convenience, the symbol “∇” will be used to represent the concatenation operation. Therefore, the input to the multimodal classifier is given by $X=\nabla_{j=1}^{J}X_{j}$, where, X is the column vector formed by concatenating the J $D_{j}$ dimensional vectors $X_{j}$. The resulting vector X will, therefore, have dimension $\left(\sum_{j=1}^{J}D_{j}\right)$. The output of the FI classifier, which is also the output of the multimodal classification system, is the K-dimensional vector Y.

### 3.1. Multimodal Feature-Integrating MLP Classifier

For this case, the network mapping can be expressed as $f:X^{D}\to Y^{K}$, where, $D=\sum_{j=1}^{J}D_{j}$ and Y is equal to $\left[P\left(\omega_{1}/X\right),P\left(\omega_{2}/X\right),\ldots ,P\left(\omega_{k}/X\right)\right]$ because of the softmax activation functions in the output layer. A multimodal test vector X can, therefore, be assigned to class $\omega_{k}$ if

(2) $$P\left(\omega_{k}/X\right)>P\left(\omega_{i}/X\right),\;\mathrm{for}\;\mathrm{all}\;i\neq k.$$

Note that the neurons in this classifier may be regarded as multisensory because the neurons in the first hidden layer receive and process features from every modality, and this information is propagated through the nodes in the subsequent layers.

### 3.2. Multimodal Feature-Integrating MAP Classifier

Because the input X to the multimodal classifier is a concatenation of Gaussian vectors, it will be assumed that X is also Gaussian. Therefore, the probability density function of X under class $\omega_{k}$ is given by

$$P\left(X/\omega_{k}\right)\sim \;G\left(\nabla_{j=1}^{J}\left(m_{j/k}\Phi_{j}S_{j/k}+c_{j/k}\right),\nabla_{i=1}^{J}\nabla_{j=1}^{J}\Psi_{ij}\right),$$

where, $\Psi_{ij}$ is the cross-covariance matrix of the signals $X_{i}$ and $X_{j}$. Note that the resulting covariance matrix with dimension D×D is a matrix of cross-covariance matrices. The rule in Equation (2) can be used to assign a test vector X to the class yielding the maximum posterior probability.

## 4. Decision-Integrating Multimodal Classification Model

**Figure 4:**
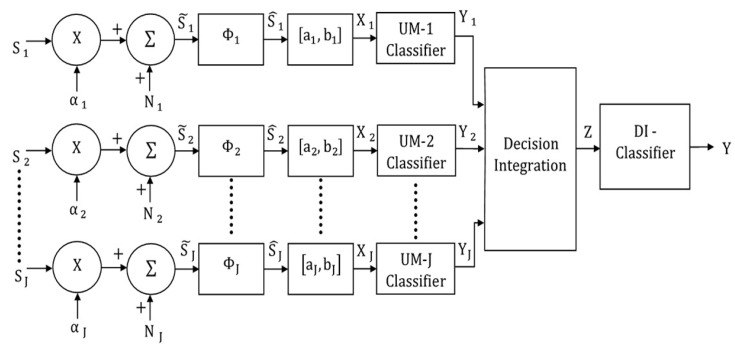
Block diagram of the decision-integrating multimodal classification system.

The decision-integrating multimodal classification model is illustrated in Figure 4. In this 2-stage classification model, the unimodal stimuli are first classified independently, and the unimodal classification results are combined through concatenation. In the next stage, the concatenated result is classified by the multimodal classifier, represented by DI, in order to determine the class of the input multimodal signal. This model can be regarded as a systems-level representation of the case in which unimodal stimuli are processed separately in the primary cortical areas and combined subsequently in multisensory association areas [16,17].

### 4.1. Decision-Integrating MLP Classifier

In this case, the J unimodal classifiers and multimodal classifier are MLPs. The input to the multimodal classifier is the concatenation of the K outputs (posterior probabilities) of the unimodal networks in the system. Therefore, the mapping for the multimodal MLP classifier can be expressed as $f:Z^{KJ}\to Y^{K}$, where, Z is the input concatenated vector with dimension (K)(J) and Y is the output with dimension K. That is,

$$Z=[P\left(\omega_{1}/Y_{1}\right),P\left(\omega_{2}/Y_{1}\right),\ldots ,P\left(\omega_{k}/Y_{1}\right),P\left(\omega_{1}/Y_{2}\right),P\left(\omega_{2}/Y_{2}\right),\ldots ,P\left(\omega_{k}/Y_{2}\right),\ldots ,\;P\left(\omega_{1}/Y_{J}\right),P\left(\omega_{2}/Y_{J}\right),\;\ldots ,\;P\left(\omega_{k}/Y_{J}\right)]$$

and $Y=\left[P\left(\omega_{1}/Z\right),P\left(\omega_{2}/Z\right),\ldots ,P\left(\omega_{k}/Z\right)\right]$.

For this case, a test multimodal stimulus is assigned to class $\omega_{k}$ where k is given by

(3) $$P\left(\omega_{k}/Z\right)>P\left(\omega_{i}/Z\right),\;for\;all\;i\neq k.$$

Note that the neurons in the unimodal classifiers are unisensory, whereas the neurons in the fusion classifier can be regarded as multisensory.

### 4.2. Decision-Integrating MAP Classifier

In this classification system, the unimodal classifiers and the multimodal classifier are MAP classifiers. The outputs (independent decisions) $Y_{j},j=1,2,\ldots ,J$ of the J unimodal MAP classifiers are the inputs to the second stage discrete MAP classifier. The probability density function of $Y_{j}$ under $\omega_{k}$ can be written as

$$P\left(Y_{j}/\omega_{k}\right)=[P_{j}{\left(\omega_{1}/\omega_{k}\right)}^{\delta \left(Y_{j}-\omega_{1}\right)}P_{j}{\left(\omega_{2}/\omega_{k}\right)}^{\delta \left(Y_{j}-\omega_{2}\right)}\ldots P_{j}{\left(\omega_{K}/\omega_{k}\right)}^{\delta \left(Y_{j}-\omega_{K}\right)}]$$

where, $P_{j}\left(\omega_{i}/\omega_{k}\right)$ is the probability that unimodal classifier j decides $\omega_{i}$ when the true class is $\omega_{k}$ and

$$\delta \left(y-\omega \right)=\{\begin{array}{c} 1,\;if\;y=\;\omega \;\\ 0,\;if\;y\neq \;\omega \end{array}.$$

If the input to the second classifier is $Z=\left(Y_{1},Y_{2},\ldots ,Y_{J}\right)$, the probability density function of *Z* can be written as

$$P\left(Z/\omega_{k}\right)=\overset{J}{\underset{j=1}{\prod }}\begin{array}{c} {}[P_{j}{\left(\omega_{1}/\omega_{k}\right)}^{\delta \left(Y_{j}-\omega_{1}\right)}P_{j}{\left(\omega_{2}/\omega_{k}\right)}^{\delta \left(Y_{j}-\omega_{2}\right)}\\ \ldots P_{j}{\left(\omega_{K}/\omega_{k}\right)}^{\delta \left(Y_{j}-\omega_{K}\right)}]. \end{array}$$

Note that Z is a J-dimensional vector, and the posterior probability of the decision-integrating classifier is given by

$$P\left(\omega_{i}/Z\right)=\frac{P\left(Z/\omega_{i}\right)P\left(\omega_{i}\right)}{\sum_{i=1}^{K}P\left(Z/\omega_{i}\right)P\left(\omega_{i}\right)},\;i=1,2\ldots K,$$

and the test multimodal stimulus is assigned to class $\omega_{k}$, where, k is given by the rule in Equation (3). 

## 5. Classification Experiments

**Figure 5:**
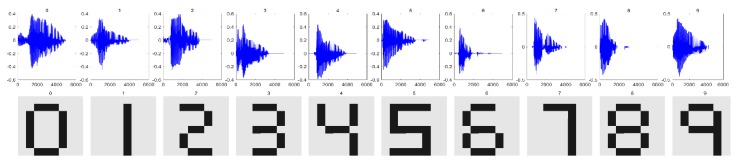
The noise-free auditory and visual stimuli used in the experiments.

Experiments were designed to demonstrate the application and evaluation of the unimodal and multimodal classifiers and to demonstrate the improvement in classification through multisensory integration. A ten-class problem involving the classification of visual and auditory signal representations of the digits 0, 1, …, 9, was considered. That is, K=10 and J=2. The visual representations were binary images generated in 64×64 arrays. The auditory signals were obtained from: evolution.voxeo.com. The adjustable sampling rate was set to 8 kHz. After start-point and end-point segmentation, the average duration of the ten noise-free auditory signals was 4000 samples. The noise-free auditory and visual signals are shown in Figure 5. For each image, the rows were concatenated to yield a vector of dimension 4096. Using the ranking method described in [27], features from the input signal $S_{j/k}$ were extracted by selecting a subset of 64 DCT coefficients. The classes were assumed to have equal a priori probabilities, that is, $P\left(\omega_{k}\right)=(1/10),\;k=1,2,\ldots ,10$.

In order to demonstrate the principle of inverse effectiveness, “weakly effective” stimuli were generated by (a) increasing the additive noise by increasing the noise variance, and (b) increasing the attenuation by decreasing the attenuation factor. The multimodal stimuli in all training sets were semantically congruent and had no attenuations (i.e., $\alpha_{j}=1,\;j=1,2$). The MLPs had three layers: an input layer, a hidden layer, and an output layer. The neural networks were trained systematically with increasingly noisy signal sets [28], and training was terminated when the cross-entropy fell below 0.001. For the MAP classifiers, the mean vectors and covariance matrices were estimated from the noisy training sets.

A total of eight classification systems were implemented: MLP and MAP implementations of the unimodal visual, unimodal auditory, and the two auditory-visual multimodal classifiers. The systems are represented by the following abbreviations: unimodal MLP for auditory stimuli (UM-MLP(A)), unimodal MLP for visual stimuli (UM-MLP(V)), unimodal MAP for auditory stimuli (UM-MAP(A)), unimodal MAP for visual stimuli (UM-MAP(V)), feature-integrating MLP system (FI-MLP), feature-integrating MAP (FI-MAP), decision-integrating MLP(DI-MLP), and decision-integrating MAP (DI-MAP). The inputs to the multimodal classifiers during training and testing were semantically congruent auditory and visual stimuli.

### 5.1. Noise Generation

**Figure 6:**
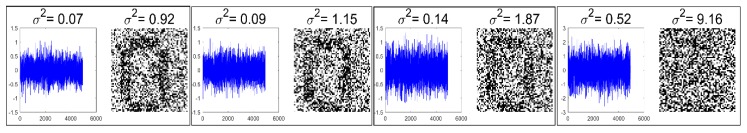
Examples of pairs of noisy auditory and visual representations of the digit 0.

**Figure 7:**
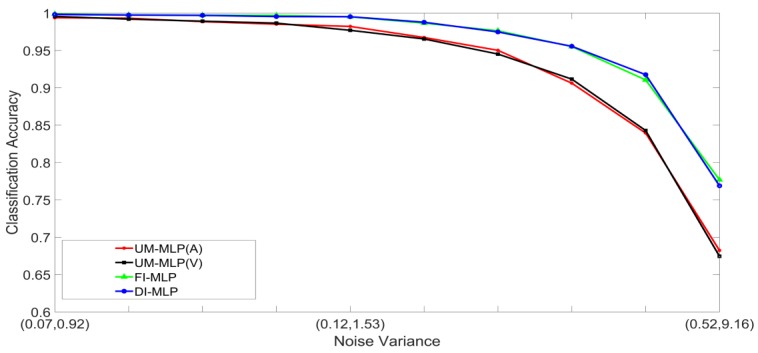
Classification accuracy, as a function of noise, for the MLP-based classification systems.

**Figure 8:**
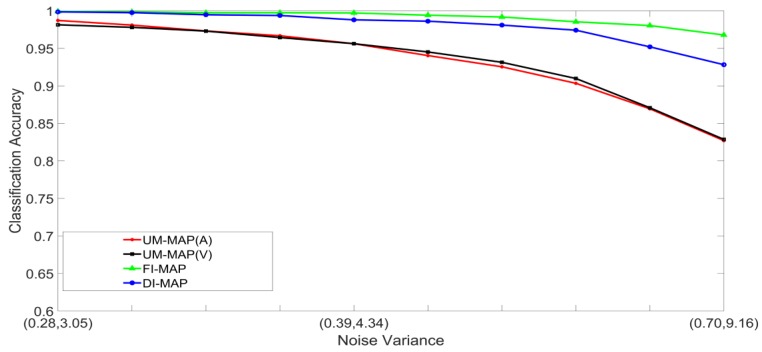
Classification accuracy, as a function of noise, for the MAP-based classification systems.

**Figure 9:**
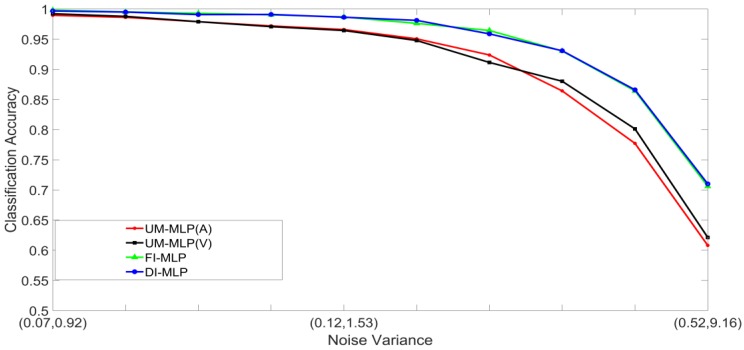
Classification accuracy, as a function of noise, and α=0.9 in both stimuli, for the MLP-based classification systems.

**Figure 10:**
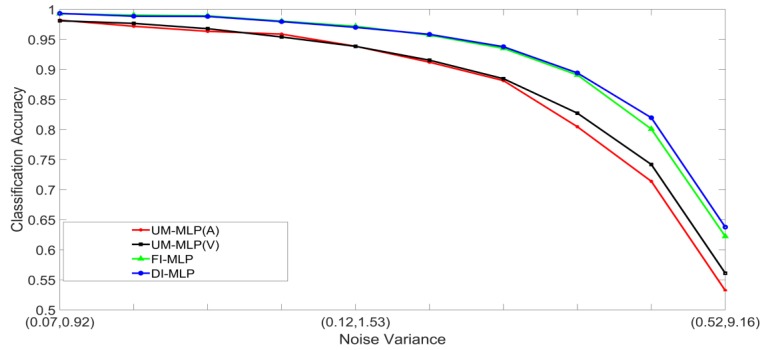
Classification accuracy, as a function of noise, and α=0.8 in both stimuli, for the MLP-based classification systems.

**Figure 11:**
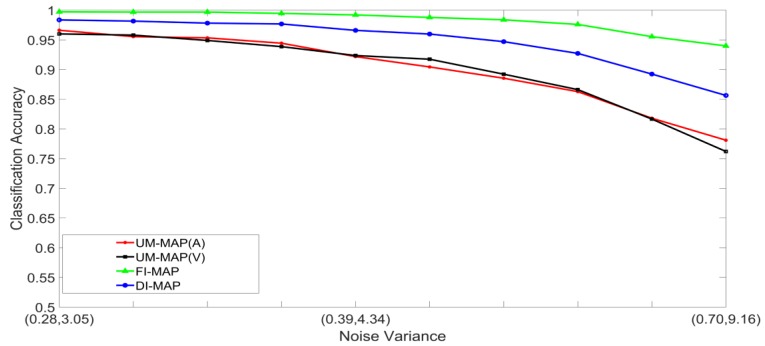
Classification accuracy, as a function of noise, and α=0.9 in both stimuli, for the MAP-based classification systems.

**Figure 12:**
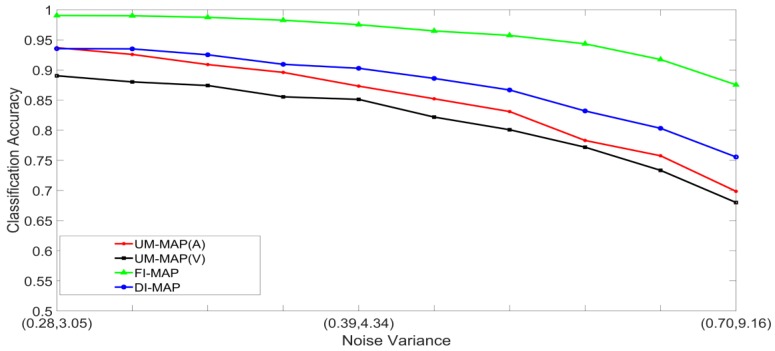
Classification accuracy, as a function of noise, and α=0.8 in both stimuli, for the MAP-based classification systems.

Noisy stimuli were generated by adding Gaussian noise drawn from $G\left(0,\sigma_{j/k}^{2}\right)$, to the noise-free input stimuli. The effects of a given noise variance on the classification accuracy varied across the different classes within a modality; therefore, the average value of $\sigma_{j/k}^{2}$ across all the classes was used for the signals in modality j. Furthermore, the effects of additive noise with a given variance varied across the two modalities. Using validation sets, noise variances which yielded approximately the same unimodal classification accuracies across the two modalities were determined and paired. The noise was, therefore, specified as a pair (A,V), where, A and V are the noise variances of the auditory and visual stimuli, respectively. Examples of four noisy auditory and visual signal pairs yielding similar MLP unimodal performances are shown in Figure 6. For brevity, only the noisy pairs for the digit 0 are shown. The effects of noise on the other digits are quite similar. This method of pairing noise variances was used to generate training and test sets for the unimodal and multimodal classifiers. A total of two hundred noisy auditory and visual stimuli were generated with varying levels of noise. The noisy stimuli were divided randomly into two equal sized sets. One set was used to train the classifier systems, and the other set was used to test the systems. For the MAP systems, training involved estimating the class mean vectors and class covariance matrices from the stimuli in the training set. For the MLP systems, training involved presenting the stimuli in the training set repeatedly until the convergence criterion was satisfied. The stimuli in the test set were classified and averaged to yield the classification accuracy at each noise-pair level. The following experiments were designed:

- Set 1: Classification of noisy stimuli—These experiments were designed to evaluate the performance of the unimodal and multimodal classification systems with noisy stimuli. The noise levels in the test stimuli were varied while the attenuation factor was set to 1. The classification accuracies, as functions of paired noise variances, are shown in Figure 7 and Figure 8.
- Set 2: Classification of noisy attenuated stimuli—These experiments were designed to evaluate the performance of the unimodal and multimodal classification systems with stimuli that were both noisy and attenuated. The experiments in Set 1 were repeated using α=0.9 and α=0.8 for both stimuli. The classification accuracies are shown in Figure 9, Figure 10, Figure 11 and Figure 12.

### 5.2. Discussion of Results

Figure 7, Figure 8, Figure 9, Figure 10, Figure 11 and Figure 12 clearly show that the classification accuracies of the multimodal classification systems are higher than those of the unimodal systems. Furthermore, multicomparison ANOVA tests conducted on the results which were used to display Figure 7 and Figure 8 confirmed that the differences between the multimodal and unimodal results were statistically significant (*p* < 0.05). Note that the noise variances used for testing the MAP classifiers are higher than those used for testing the MLP classifiers. It could, therefore, be concluded that, in these sets of experiments, the MAP classifiers perform better than the MLP classifiers. Furthermore, the FI-MAP classifiers perform better than the DI-MAP classifiers.

For this study, it is important to note that the differences between the unimodal and multimodal classification accuracies as well as the trends are more important than the actual classification accuracies. Recall that no attempt was made to select the best feature sets nor the best classifiers. Therefore, if the goal is to improve the performance of a multimodal classifier, it can be achieved by selecting better feature sets and classifiers for each modality. Although the main focus was to demonstrate the inverse effectiveness principle through the inclusion of random noise and an amplitude attenuation factor during the testing phase, the spatial and temporal rules can also be demonstrated in a similar manner by including spatial-offset and temporal-offset attenuation factors, respectively, into the models during testing. For these two cases, the attenuation factors can be regarded as penalties which increase when the distances and time intervals between the stimuli increase. Another possibility for demonstrating the temporal rule is to impose a penalty by dropping $\nabla_{j}$ samples from a delayed stimulus vector, where, $\nabla_{j}$ is proportional to the delay in the stimulus of modality j.

Finally, it could be argued that the improvement in performance of the multimodal classifiers over the unimodal classifiers should be expected because the additional congruent information available should facilitate classification. A similar argument can be made to explain the improvement in recognition in the brain due to the integration of congruent multimodal information. What is important to note, however, is that we have identified and solved key problems that need to be tackled in order to develop multimodal classifier models. These key problems include: (a) developing structures inspired by the two commonly accepted models for multisensory integration in the brain, (b) simulating the influence of stimuli and the effects of stimulus noise, (c) enabling the extraction of different stimulus representations, and (d) defining and combining information in different ways.

## 6. Conclusions

This paper focused on the development of feature-integrating and decision-integrating object classification models, both inspired by multisensory integration in the brain. The models were quite general in the sense that the classifiers were not pre-specified and the number of modalities and stimuli classes were variable. Furthermore, the models included the ability to add uncertainty to stimuli through random noise and variable attenuation levels. Consequently, the models enable the simulation and evaluation of various combinations of multimodal stimuli and classifiers under varying uncertainty conditions. The results from several sets of experiments clearly demonstrated the improvement in classification through the integration of auditory and visual stimuli, and it was noted that the models offer the potential to improve performance by selecting more effective features and classifiers for each modality.
